# *IL17F*: A Possible Risk Marker for Spondyloarthritis in HLA-B*27 Negative Brazilian Patients

**DOI:** 10.3390/jpm11060520

**Published:** 2021-06-07

**Authors:** Janisleya Silva Ferreira Neves, Jeane Eliete Laguila Visentainer, Denise Manjurma da Silva Reis, Marco Antonio Rocha Loures, Hugo Vicentin Alves, Joana Maira Valentini Zacarias, Ana Maria Sell

**Affiliations:** 1 Post Graduation Program in Biosciences and Physiopathology, Department of Clinical Analysis and Biomedicine, Maringá State University, Paraná 87030-900, Brazil; janegarcia1003@gmail.com (J.S.F.N.); jelvisentainer@uem.br (J.E.L.V.); de_manjurma@hotmail.com (D.M.d.S.R.); mloures@gmail.com (M.A.R.L.); hugovicentin@gmail.com (H.V.A.); anamsell@gmail.com (A.M.S.); 2 Post Graduation Program in Biosciences and Physiopathology, Department of Clinical Analysis and Biomedicine and Department of Basic Health Sciences, Maringá State University, Paraná 87030-900, Brazil; 3 Department of Medicine, Maringa State University, Paraná 87030-900, Brazil

**Keywords:** genetic association studies, ankylosing spondylitis, psoriatic arthritis, HLA-B27 antigen, *IL17F C* variant

## Abstract

HLA-B*27 is an important marker for spondyloarthritis (SpA), however, many SpA patients are HLA-B*27 negative. Thus, the aim of this study was to investigate the influence of *IL17*, *TNF* and *VDR* gene polymorphisms in SpA patients who were HLA-B*27 negative. This case-control study was conducted in 158 patients [102 patients with ankylosing spondylitis (AS) and 56 with psoriatic arthritis (PsA)] and 184 controls. HLA-B*27 genotyping was performed using PCR-SSP and *IL17A* (rs2275913), *IL17F* (rs763780), *TNF-308* (rs1800629), *TNF-238* (rs361525), *FokI* C>T (rs2228570), *TaqI* C>T (rs731236), *ApaI* A>C (rs7975232), and *BsmI* C>T (rs1544410) using PCR-RFLP. Statistical analyses were performed by Chi-square and logistic regression using OpenEpi and SNPStats software. The *IL17F C* allele frequency was higher in patients with SpA, AS and PsA compared to controls. The *IL17F T/C* genotype frequency was higher in SpA patients in an overdominant inheritance model and when men and women were separately analyzed. *IL17A_IL17F AC* haplotype was significantly associated to the risk for SpA patients. As for *VDR*, the *ApaI a/a* was a potential risk factor for SpA in men. In conclusion, *IL17F C* variant contributed to the risk of SpA in Brazilian patients who were HLA-B*27 negative and could be a potential marker for SpA.

## 1. Introduction

Spondyloarthritis (SpA) is a group of autoimmune and rheumatic disorders with a family pattern, affecting the axial skeleton, peripheral joints and entheses, and with extraskeletal site manifestations. SpA is divided into subtypes: ankylosing spondylitis (AS), reactive arthritis (ReA), psoriatic arthritis (PsA), arthritis related to inflammatory bowel disease (EI) and undifferentiated SpA. Ankylosing spondylitis is a chronic immune-mediated disease and is the most frequent form of SpA. The inflammation predominantly affects the axial skeleton; however, peripheral arthritis and enthesopathy are present in many AS patients [1]. PsA is a chronic, inflammatory and musculoskeletal disease associated with psoriasis. It manifests in peripheral arthritis, dactylitis, enthesitis and spondylitis [2].

HLA-B27 antigens are considered to be of major importance in the pathogenesis of the disease and with a strong genetic susceptibility to SpA, contribute to almost 20% of the heritability for AS. However, only a minority of HLA-B*27 carriers develop the disease (1–5%) [1] and individuals who lack the HLA-B*27 alleles also develop SpA. In addition, other non-HLA genes have been associated with the risk of SpA [3,4]. The mechanism by which the HLA-B27 is predisposed to spondyloarthritis remains unresolved [4] and arthritogenic peptides have not been defined in humans and are not involved in experimental models of spondyloarthritis [5]. The pathogenesis theories for SpA include, in addition to the biochemical properties of HLA-B27, the production of tumor necrosis factor-α (TNF-α), interleukin-17 (IL-17), IL-23 and interferon-γ [5,6,7,8]. In rodent models independent of HLA-B27, spondyloarthritis can be driven by CD4-/CD8- T resident cells in entheses and triggered by IL-23 and by Th17 cells [5].

Increased SpA activity and structural damage, especially regarding entheses inflammation and ossification, is associated with Vitamin D deficiency [9]. The modulating effects of vitamin D are mediated by the *VDR* (Vitamin D Receptor gene), which participates by suppressing the autoimmune processes and tissue damage and delaying chronic disease by inhibiting T helper 1 (Th1) and 17 (Th17) immune response [10,11]. A possible interaction of cytokines and the vitamin D system could define new diagnostic and therapeutic implications in spondyloarthritis, mainly in HLA-B*27 negative patients. 

Previous studies carried out in our laboratory have shown the association of *VDR Apa*I and *Fok*I polymorphisms with PsA [12], and *TNF* and *IL17* variants, which influence the good production of the respective cytokines, were associated with SpA, AS and PsA regardless of gender and HLA-B*27 [13]. In both, the HLA-B*27 was considered a covariate in multivariate analysis, but patients who were negative for HLA-B*27 were not investigated. 

Thus, the aim of this study was to investigate the influence of *IL17, TNF* and *VDR* gene polymorphisms in SpA patients who were HLA-B*27 negative. The correlation between these polymorphisms with BASDAI (Bath Ankilosing Spondilitis Disease Activity Index) and vitamin D deficiency was also analyzed. We were able to observe from this investigation that *IL17F C* variant contributed to the risk of SpA in Brazilian patients who were HLA-B*27 negative and could be a potential marker for SpA. The elucidation of the role of this cytokine in pathogenesis of the disease could define new diagnostic and therapeutic implications in spondyloarthritis.

## 2. Materials and Methods

### 2.1. Sample Selection

The Research and Ethics Committee of the State University of Maringá (number CA/AE 27723114) approved the study, and everyone who agreed to participate signed the consent form. This is a case-control study conducted in HLA-B*27 negative individuals, consisting of 158 patients with spondyloarthritis (SpA) and 184 controls; among them, 102 had ankylosing spondylitis (AS) and 56 had psoriatic arthritis (PsA). All individuals had follow-ups with the same rheumatologists from the University Hospital of Maringá. The disease diagnosis was performed through clinical, laboratory and radiological criteria according to the ASAS 2009/2011 criteria for axial and for peripheral SpA [14,15]. Patients and controls were matched by ethnicity, gender and age, and were not related. Some patients had BASDAI (N = 66) and serum concentration levels for hydroxyl (OH) vitamin D (N = 40) performed before starting treatment, and all of them did not have vitamin D replacement at the time of diagnosis. The non-inclusion criteria for all samples included individuals with diabetes, inflammatory diseases, autoimmunity and chronic diseases. All participants were from the northwestern region of Paraná, in southern Brazil (22°29′30″–26°42′59″ S and 48°02′24″–54°37′38″ W) of predominantly European origin and classified as mixed ethnicity, as previously defined for the population of this Brazilian region [16,17], and Asian descendants were not included in the sample.

### 2.2. Technical Procedures

The blood was collected in vacuum tubes with EDTA, DNA was obtained using the salting-out method [18] and DNA’s concentration and quality were analyzed using optical density in Nanodrop 2000^®^ (ThermoScientific-Wilmington, DE, USA). 

HLA-B*27 genotyping was performed using PCR-SSP [19] and *IL-17A* (rs2275913), *IL-17F* (rs763780), *TNF-308* (rs1800629) and *TNF-238* (rs361525) genotyping was performed using PCR-RFLP according to the methods previously described and validated in our laboratory [13,20]. Genotyping for *VDR* SNPs was conducted as previously described for *FokI* C>T (rs2228570) [21] and for *Taq*I T>C (rs731236), *Apa*I A>C (rs7975232) and *Bsm*I C>T (rs1544410) [22] as previously validated for our population [23]. The quality of the genotyping was guaranteed by direct sequencing of 15 samples for each SNP: for SBT, the kits were optimized to run with Dye Set E for Brilliant Dye Terminator v1.1 and Dye Set Z for Brilliant Dye Terminator v3.1. We found 100% agreement between the two methods.

BASDAI was performed according to criteria previously defined [24] by the same rheumatologists. The scores were defined from the visual analogical scale as 0 to 10 (0 = good; 10 = bad): scores <4.0 indicated good clinical activity and ≥4.0 high clinical activity.

Serum concentration of the 25-hydroxy vitamin D3 (25OH) was determined using the chemiluminescence technique (Abbot/t reagent Ireland Diagnostics Division, Lisnamuck-Longfor, Ireland), following manufacturer’s recommendations.

### 2.3. Statistical Analysis

The association of gene polymorphisms with the SpA, SA and PsA was carried out using logistic regression and Chi-square analysis. The *p*-value was considered statistically significant when less than 5% and, for these, the odds ratios and their respective 95% confidence intervals (CI) were defined. For these analyses and for estimates the distribution of genotype frequencies according to the Hardy-Weinberg equilibrium (HWE), OpenEpi Version 3.01 (https://www.openepi.com/Menu/OE_Menu.htm, accessed on: 31 January 2021) and SNPStats software (https://www.snpstats.net/start.htm, accessed on: 31 January 2021) [25] were applied. The better inheritance model of association (codominant, dominant, recessive, overdominant and log-additive) was chosen using the lower Akaike information criterion (AIC) [25]. Covariate analysis included gender, BASDAI and Vitamin D serum concentration. Continuous data were given as mean ± standard deviation (OpenEpi Version 3.01). The Bonferroni adjustment for multiple testing was applied and the corrected value (*Pc*) was obtained after the multiplication of the *p*-value by the number of analyzed SNPs (five SNPs considering those in linkage disequilibrium (*p* < 0.001): *IL17A-IL17F* (∆′ = 0.38), *Taq*I*-Apa*I (∆′ = 0.63), *Taq*I*-Bsm*I (∆′ = 0.49). Bootstrapping by Haploview software Version 4.2 was performed to assess the robustness of the results [26]. In order to obtain the minimum number of samples adequate for carrying out this study and with adequate statistical power (≥80%), the quantitative calculation software QUANTO (www.biostats.usc.edu/software, accessed on: 31 January 2021) was used. For this, we consider the less frequent allele (0.095 for *TNF*-238), population risk (1.5%), and OR (2.0–4.0).

## 3. Results

The characteristics of HLA-B*27 negative patients (SpA, AS and PsA) and HLA-B*27 negative controls, distributed according to gender, age, BASDAI and vitamin D sufficiency are shown in Table 1. Patients and controls were matched according to age and gender. SpA mean age was 49.36 (±16.04) and for control 40.92 (±12.16) years old, which was similar for AS and PsA. Males were 40.50% of the patients and 41.85% of the controls, which was similar for AS and PsA.

The distribution of the genotype frequencies for all analyzed genes in the control group was consistent with the Hardy-Weinberg equilibrium (*p* > 0.05). The distribution of the allele frequencies for all analyzed genes in the control group was in line with what was expected for Brazilians in the northwestern region of Paraná (for *TNF*, *IL17A,* and *IL17F*: http://www.allelefrequencies.net, accessed on: 31 January 2021 and Reis et al., 2017 [27]; for *VDR:* Pepineli et al., 2019 [23]).

The allele frequency of the *IL17F C* variant was higher in SpA, AS, and PsA patients compared to controls (*Pc* < 0.015 and OR= 3.39, 2.60, and 4.93, respectively). In addition, the *IL17F T/C* genotype frequency was higher in SpA, AS and PsA compared to control in codominant, dominant, overdominant and log-additive inheritance models. The inheritance model of choice according to AIC was overdominant (*Pc* <0.001; OR = 4.31, 3.36, and 8.33, respectively for SpA, AS and PsA). In an overdominant inheritance model, the heterozygous was compared to a pool of both homozygous alleles; in other words, *T/C* was compared to *T/T+C/C*.

The *IL17A A/A* genotype frequency was higher in PsA patients when compared with controls in a recessive inheritance model, however the significance was lost after Bonferroni correction. As for *VDR*, the *Apa*I *a/a* genotype frequency was higher in AS patients compared to controls in a recessive model, but significance was lost after Bonferroni correction. No differences were found in the distribution of genotype and allele frequencies for *TNF* and other *VDR* polymorphisms, when SpA, AS and PsA patients were compared with controls. Results are shown in Table 2.

*IL17A_IL17 AC* haplotype frequency was higher in SpA patients (*Pc* = 0.001, OR = 9.93, 95% CI = 2.93–33.59); this haplotype is related to the good production of IL-17 cytokines.

SpA is linked to males, however, this disease is an important cause for disability in females [28,29,30], and thus gender was considered in association analyses. Differences in the genotype frequency distributions of *IL17F* and *Apa*I were observed in male and in female HLA-B*27 negative patients with SpA when compared with same-sex controls (Table 3). *IL17F T/C* genotype frequency was higher in women and in men with SpA (*Pc* < 0.004, OR = 6.41, and *Pc* = 0.05, OR = 3.29, respectively) than controls. The genotype frequency of *VDR Apa*I *a/a* was higher in men with SpA than in controls (*Pc* < 0.02, OR = 3.04).

Few patients had BASDAI and vitamin D concentrations determined at the time of diagnosis (no vitamin D therapeutic intervention). Nevertheless, we analyzed whether the *IL17* and *TNF* genotypes were associated with SpA in patients with better clinical disease activity and vitamin D sufficiency (Table 4). In patients with SpA the frequency of the genotypes related to the lower pro-inflammatory cytokine production (*IL17A G/G* and *TNF-308 G/G*) were higher in those with vitamin D sufficiency and BASDAI ≤ 4.0.

## 4. Discussion

In spondyloarthritis (SpA), the immunological involvement and the familial pattern characterize a strong genetic participation in the pathogenesis. HLA-B*27 is the main disease marker in SpA, but genome-wide association studies have identified new associations between polymorphisms in genes with an immunological function, particularly in genes that control the interleukin (IL)-23/IL-17 signaling pathway. In addition, the effectiveness of IL-17 inhibitors in the treatment of patients with SpA highlights the impact of this pathway on the disease [1,31].

Research conducted by our group showed that *VDR, TNF* and *IL17* polymorphisms were associated to the SpA [12,13]. The association of these polymorphisms in patients with absence of HLA-B*27 was not performed and, in this study, we assessed whether the polymorphisms in *IL17, TNF* and *VDR* genes influence the development of SpA in patients who were HLA-B*27 negative.

The main finding of this study was that *IL17F C* allele was associated with the risk for SpA, AS and PsA in HLA-B*27 negative patients. In addition, the *IL17F T/C* genotype and the *IL17A_IL17F AC* haplotype, both associated with good IL-17 production, were associated to SpA in HLA-B*27 negative patients. The *IL17F* *T/C* genotype had a significant association to the SpA in HLA-B*27 negative in both men and women.

The *IL17F* rs763780 *C* allele has been associated to an increased risk of autoimmune and inflammatory diseases such as rheumatoid arthritis in Tunisian population [32], inflammatory arthritis in a meta-analysis conducted in Caucasians [33], hip osteoarthritis in Han Chinese [34], knee osteoarthritis in Iranians [35], and psoriasis an Asians [36]. Regarding SpA, the *IL17F* polymorphism was associated with susceptibility to AS, disease activity and functional status in Turk patients [37], and a risk factor for SpA, AS and PsA in Brazilians [13]. According to Bafrani et al., 2019 [35] the *IL17* polymorphisms can be considered biomarkers for the screening of knee osteoarthritis susceptible persons.

IL-17 is a pro-inflammatory cytokine consisting of six structurally related cytokines. The IL-17A and IL-17F are the most closely related, co-expressed in linked genes, and usually co-produced by Type 17 cells [38]. IL-17 is a critical mediator of inflammation and has synergistic ability with other inflammatory signs, which makes it a vital inflammatory effector [39]. This cytokine also promotes osteogenesis, mainly at inflamed sites undergoing mechanical stress, such as entheses [40].

The association of HLA-B27 with spondyloarthritis varies markedly among different SpA and among different ethnic groups [41,42,43,44]. HLA-B*27, generally present in about 90% of patients with AS in non-mixed populations, was positive in 50% to 70% of the Brazilian and Ibero-American patients [43,44]. The lower frequency of HLA-B*27 in SpA in populations with a higher degree of heterogeneity as in Latin America and the complexity of the immunological pathways that determine the predisposition and phenotypic variations of SpA, justify the search for new markers that help in the diagnosis of the disease in mixed populations and our study in Brazilians.

Although we previously reported that *TNF* polymorphisms were related to the risk for SpA [13], in this study conducted with patients with SpA who were HLA-B*27 negative, no association was found between *TNF*-308 (rs1800629) and *TNF*-238 (rs361525) and disease.

*VDR* polymorphisms can modify the transcription of mRNA and influence the development of SpA. Previously we found that *Apa*I *a/a* was a protective factor for PsA and a risk for AS in men [12]. In this study we also found that *VDR Apa*I *a/a* genotype was a risk factor for men with SpA who were HLA-B*27 negative.

Vitamin D deficiency is a predisposing factor for SpA [4,45], and has been associated with increased activity of axial disease in spondyloarthritis [46,47] and with more prominent symptoms, such as pain inflammation and structural damage due to less bone activity [9,48,49]. Thus, considering the vitamin D sufficiency and the clinical activity of the disease, we investigate if *IL17*, *TNF* and *VDR* polymorphisms were associated to SpA. Because vitamin D deficiency is pandemic, it was difficult to select patients without hormonal therapy and, therefore, the number of HLA-B*27 negative patients classified according to BASDAI and without Vitamin D therapy was small. Even so, important results were found: genotypes related to lower proinflammatory cytokine production (IL-17F and TNF-α) were associated to the better clinical conditions of SpA in HLA-B*27 negative patients with sufficiency of vitamin D.

Several other HLA-B and non-HLA-B molecules, in addition to HLA-B27, have been associated with the SpA and considerable progress has been made in understanding the complex immunopathogenesis of the disease [50]. The danger signals released by innate immune cells activate Th1 and Th17 lymphocytes leading to inflammation, synovitis, enthesitis and altered bone homeostasis; cytokines, such TNF-α, IL-17, IL-23, IL-1, and interferon-γ participate in these immunological mechanisms [51,52]. Knowledge on genetics and immunology has improved treatment options with the availability of treatments targeting tumor necrosis factor-α (TNF-α) and interleukin (IL)-17 [31,53]. Our findings are consistent with the immunological pathway of IL-23/IL-17, which plays an important role in promoting the onset and perpetuation of the SpA.

To our knowledge, this is the first study that assesses the possibility of including *IL17F* C allele as a possible biological marker in SpA, in addition to HLA-B*27. This fact is particularly relevant considering that this could be a possible new tool to help SpA diagnosis in mixed populations whose frequencies of HLA-B*27 in the disease are lower than in Caucasians.

## 5. Conclusions

*IL17F* variant confers susceptibility for SpA, AS and PsA in Brazilian patients who were HLA-B*27 negative and the *IL17 C* allele could be a potential marker for SpA in mixed populations. The elucidation of the role of other non-HLA molecules in the immunopathogenesis of the disease could define new diagnostic and therapeutic implications in spondyloarthritis.

## Figures and Tables

**Table 1:** Characteristics of HLA-B*27 negative patients with spondyloarthritis (SpA), ankylosing spondylitis (AS), and psoriatic arthritis (PsA) and HLA-B*27 negative controls.

Variable	SpA	AS	PsA	Controls
	N = 158	N = 102	N = 56	N = 184
Mean age ± SD (year)	49.36 (±16.04)	45.57 (±16.06)	54.83 (±14.33)	40.92 (±12.16)
Male *n* (%)	64 (40.50)	40 (39.22)	24 (42.86)	77 (41.85)
Male mean age ± SD (year)	50.63 (±15.73)	47.30 (±16.09)	56.17 (±13.70)	40.88 (±11.63)
BASDAI	N = 66			
<4.0	23 (34.84%)			
≥4.0	43 (65.16%)			
Vitamin D	N = 40			
<29.9 ng/mL	27 (67.50%)			
>30.0 ng/mL	13 (32.50%)			

N = total number of individuals; SD = standard deviation; BASDAI: Bath Ankylosing Spondylitis Disease Activity Index.

**Table 2:** Distribution of the allele and genotype frequencies of *IL17A, IL17F*, *TNF*-238, *TNF*-308, *Fok*I, *Taq*I, *Apa*I, and *Bsm*I in HLA-B*27 negative patients with SpA, AS, and PsA and controls.

Genotypes and Alleles	SpA	AS	PsA	Control	OR (95% CI)	*p*-Value	*Pc*
	n (f)	n (f)	n (f)	n (f)			
*IL17A*	N = 156 *	N = 100 *	N = 56	N = 182 *			
*G/G*	79 (0.51)	55 (0.55)	24 (0.43)	102 (0.56)			
*G/A*	60 (0.38)	38 (0.38)	22 (0.39)	67 (0.37)			
*A/A*	17 (0.11)	7 (0.07)	10 (0.18)	13 (0.07)			
Recessive *G/G+A/G* = Ref			46 (0.82)	169 (0.93)			
*A/A*			10 (0.18) ^c^	13 (0.07)	2.89 (1.18–7.08) ^c^	0.02	0.10
*G*	218 (0.70)	148 (0.74)	70 (0.62)	271 (0.74)			
*A*	94 (0.30)	52 (0.26)	42 (0.38)	93 (0.26)			
*IL17F*	N = 156	N = 100	N = 56	N = 184			
*T/T*	110 (0.71)	77 (0.77)	33 (0.59)	167 (0.91)			
*T/C*	45 (0.28)	22 (0.22)	23 (0.41)	15 (0.08)			
*C/C*	1 (0.006)	1 (0.01)	0 (0.00)	2 (0.01)			
Overdominant *T/T+C/C* = Ref.	111 (0.71)	78 (0.78)	33 (0.59)	169 (0.92)			
*T/C*	45 (0.29) ^a^	22 (0.22) ^b^	23 (0.41) ^c^	15 (0.08)	4.31 (2.32–8.01) ^a^ 3.36 (1.63–6.91) ^b^ 8.33 (3.88–17.85) ^c^	<10^−4^ <10^−4^ <10^−4^	<0.001 <0.001 <0.001
*T*	265 (0.85)	176 (0.88)	89 (0.79)	349 (0.95)			
*C*	47 (0.15)	24 (0.12)	23 (0.21)	18 (0.05)	3.39 (1.92–5.97) ^a^ 2.61 (1.38–4.93) ^b^ 4.93 (2.55–9.55) ^c^	<10^−4^ 0.003 <10^−4^	<0.0001 0.015 <0.0001
*TNF-238*	N = 156	N = 100	N = 56	N = 182			
*G/G*	130 (0.83)	88 (0.88)	42 (0.75)	155 (0.85)			
*G/A*	25 (0.16)	12 (0.12)	13 (0.23)	26 (0.14)			
*A/A*	1 (0.006)	0 (0.00)	1 (0.18)	1 (0.006)			
*G*	285 (0.91)	188 (0.94)	97 (0.87)	336 (0.92)			
*A*	27 (0.09)	12 (0.06)	15 (0.13)	28 (0.08)			
*TNF-308*	N = 156	N = 100	N = 56	N = 182			
*G/G*	106 (0.68)	68 (0.67)	38 (0.68)	119 (0.65)			
*G/A*	48 (0.31)	31 (0.31)	17 (0.30)	61 (0.34)			
*A/A*	2 (0.01)	1 (0.01)	1 (0.02)	2 (0.01)			
*G*	260 (0.83)	167 (0.84)	93 (0.83)	299 (0.82)			
*A*	52 (0.17)	33 (0.16)	19 (0.17)	65 (0.18)			
*Fok*I	N = 158	N = 102	N = 56	N = 184			
*F/F*	62 (0.39)	41 (0.40)	21 (0.38)	82 (0.45)			
*F/f*	81 (0.51	49 (0.48)	32 (0.57)	85 (0.46)			
*f/f*	15 (0.10)	12 (0.12)	3 (0.05)	17 (0.09)			
*F*	205 (0.65)	131 (0.64)	74 (0.66)	249 (0.68)			
*f*	111 (0.35)	73 (0.36)	38 (0.34)	119 (0.32)			
*Taq*I	N = 158	N = 102	N = 56	N = 184			
*T/T*	61 (0.39)	38 (0.37)	23 (0.41)	74 (0.40)			
*T/t*	77 (0.49)	52 (0.51)	25 (0.45)	89 (0.48)			
*t/t*	20 (0.13)	12 (0.12)	8 (0.14)	21 (0.12)			
*T*	199 (0.63)	128 (0.63)	71 (0.63)	237 (0.64)			
*t*	117 (0.37)	76 (0.37)	41 (0.37)	131 (0.36)			
*Apa*I	N = 158	N = 102	N = 56	N = 184			
*A/A*	52 (0.33)	29 (0.28)	23 (0.41)	57 (0.31)			
*A/a*	73 (0.46)	45 (0.44)	28 (0.50)	96 (0.52)			
*a/a*	33 (0.21)	28 (0.27)	5 (0.09)	31 (0.17)			
Recessive: *A/A-A/a* = Ref		74 (0.73)		153 (0.83)			
*a/a*		28 (0.27) ^b^		31 (0.17)	1.88 (1.05-3.36) ^b^	0.035	0.175
*A*	177 (0.56)	103 (0.50)	74 (0.66)	210 (0.57)			
*a*	139 (0.44)	101 (0.50)	38 (0.34)	158 (0.43)			
*Bsm*I	N = 158	N = 102	N = 56	N = 184			
*b/b*	55 (0.35)	34 (0.33)	21 (0.38)	70 (0.38)			
*B/b*	81 (0.51)	54 (0.53)	27 (0.48)	89 (0.48)			
*B/B*	22 (0.14)	14 (0.14)	8 (0.14)	25 (0.14)			
*b*	191 (0.60)	122 (0.60)	69 (0.62)	229 (0.62)			
*B*	125 (0.40)	82 (0.40)	43 (0.38)	139 (0.38)			

SpA: spondyloarthritis; AS: ankylosing spondylitis; PsA: psoriatic arthritis. ^a^ SpA vs. control; ^b^ AS vs. control; ^c^ PsA vs. control. *Pc*: *p*-value after Bonferroni correction. f: allele or genotype frequency. * Some samples were lost in SNP genotyping.

**Table 3:** Association of *IL17F* and *Apa*I polymorphisms with spondyloarthritis (SpA) in HLA-B*27 negative patients compared with same-sex controls.

		SpAn (f)	Controlsn (f)	*p*-Value	*Pc*	OR (95% CI)
*IL17F*		N = 156	N = 182			
female						
	*T/T*	62 (0.67)	96 (0.91)			Ref.
	*T/C*	29 (0.31)	7 (0.067)	10^−5^	0.0001	6.41 (2.65-15.54)
	*C/C*	1 (0.01)	2 (0.02)			
male						
	*T/T*	48 (0.75)	69 (0.90)			Ref.
	*T/C*	16 (0.25)	8 (0.09)	0.011	0.05	3.29 (1.26-8.59)
	*C/C*	0	0			
*Apa*I		N = 158	N = 182			
male						
	*A/A*	19 (0.30)	26 (0.34)			Ref.
	*A/a*	25 (0.39)	42 (0.55)			
	*a/a*	20 (0.31)	9 (0.12)	0.004	0.02	3.04 (1.14-8.14)

*Pc*: *p*-value after Bonferroni correction. f: genotype frequency.

**Table 4:** Association of *IL17* and *TNF* polymorphisms with SpA HLA-B*27 negative patients evaluated according to BASDAI and vitamin D concentration.

		Spondyloarthritis (SpA) N = 66			
Genotype	Vitamin D	BASDAI < 4.0	BASDAI ≥ 4.0	*p*-Value	OR (95% CI)
		n (f)	n (f)		
*IL17A G/G*	Deficiency	3 (0.20)	6 (0.67)		
	Sufficiency	12 (0.80)	3 (0.33)	0.03	0.12 (0.02–0.82)
*TNF-308 G/G*	Deficiency	3 (0.12)	9 (0.42)		
	Sufficiency	20 (0.88)	12 (0.57)	0.03	0.20 (0.05–0.89)

Vitamin D sufficiency: serum concentration >30 ng/mL. f: genotype frequency. BASDAI: Bath Ankylosing Spondylitis Disease Activity Index. All genotypes of *IL17A, IL17F*, *TNF*-238, *TNF*-308, *Fok*I, *Taq*I, *Apa*I, and *Bsm*I were analyzed, but only significant results were shown.

## Data Availability

The data that support the results described in this work are available in this manuscript. However, if further details are needed, the authors are available to provide the data.

## References

[1] Cortes A., Hadler J., Pointon J.P., Robinson P.C., Karaderi T., Leo P., Cremin K., Pryce K., Harris J., Lee S. (2013). Identification of multiple risk variants for ankylosing spondylitis through high-density genotyping of immune-related loci. Nat. Genet..

[2] Singh J.A., Guyatt G., Ogdie A., Gladman D.D., Deal C., Deodhar A., Dubreuil M., Dunham J., Husni M.E., Kenny S. (2019). Special Article: 2018 American College of Rheumatology/National Psoriasis Foundation Guideline for the Treatment of Psoriatic Arthritis. Arthritis Care Res..

[3] Duffin K.C., Chandran V., Gladman D.D., Krueger G.G., Elder J.T., Rahman P. (2008). Genetics of psoriasis and psoriatic arthritis: Update and future direction. J. Rheumatol..

[4] Akkoç N., Yarkan H., Kenar G., Khan M.A. (2017). Ankylosing Spondylitis: HLA-B*27-Positive Versus HLA-B*27-Negative Disease. Curr. Rheumatol. Rep..

[5] Colbert R.A., Navid F., Gill T. (2017). The role of HLA-B*27 in spondyloarthritis. Best Pract. Res. Clin. Rheumatol..

[6] DeLay M.L., Turner M.J., Klenk E.I., Smith J.A., Sowders D.P., Colbert R.A. (2009). HLA-B27 misfolding and the unfolded protein response augment interleukin-23 production and are associated with Th17 activation in transgenic rats. Arthritis Rheum..

[7] Pedersen S.J., Maksymowych W.P. (2018). Beyond the TNF-α Inhibitors: New and Emerging Targeted Therapies for Patients with Axial Spondyloarthritis and their Relation to Pathophysiology. Drugs.

[8] Pedersen S.J., Maksymowych W.P. (2019). The Pathogenesis of Ankylosing Spondylitis: An Update. Curr. Rheumatol. Rep..

[9] Guillot X., Prati C., Wendling D. (2014). Vitamin D and spondyloarthritis. Expert Rev. Clin. Immunol..

[10] Tang J., Zhou R., Luger D., Zhu W., Silver P.B., Grajewski R.S., Su S.-B., Chan C.-C., Adorini L., Caspi R.R. (2009). Calcitriol Suppresses Antiretinal Autoimmunity through Inhibitory Effects on the Th17 Effector Response. J. Immunol..

[11] Joshi S., Pantalena L.-C., Liu X.K., Gaffen S.L., Liu H., Rohowsky-Kochan C., Ichiyama K., Yoshimura A., Steinman L., Christakos S. (2011). 1,25-Dihydroxyvitamin D3 Ameliorates Th17 Autoimmunity via Transcriptional Modulation of Interleukin-17A. Mol. Cell. Biol..

[12] Neves J.S.F., Visentainer J.E.L., da Reis D.M.S., Rocha Loures M.A., Alves H.V., Lara-Armi F.F., De Alencar J.B., Valentin Zacarias J.M., Sell A.M. (2020). The Influence of Vitamin D Receptor Gene Polymorphisms in Spondyloarthritis. Int. J. Inflam..

[13] Rocha Loures M.A., Macedo L.C., Reis D.M., Oliveira C.F., Meneguetti J.L., Martines G.F., Neves J.S.F., de Souza E., Sell A.M., Visentainer J.E.L. (2018). Influence of *TNF* and *IL17* Gene Polymorphisms on the Spondyloarthritis Immunopathogenesis, Regardless of HLA-B27, in a Brazilian Population. Mediators Inflamm..

[14] Sieper J., Rudwaleit M., Baraliakos X., Brandt J., Braun J., Burgos-Vargas R., Dougados M., Hermann K.G., Landewe R., Maksymowych W. (2009). The Assessment of SpondyloArthritis international Society (ASAS) handbook: A guide to assess spondyloarthritis. Ann. Rheum. Dis..

[15] Rudwaleit M., Van Der Heijde D., Landewé R., Akkoc N., Brandt J., Chou C.T., Dougados M., Huang F., Gu J., Kirazli Y. (2011). The Assessment of SpondyloArthritis international Society classification criteria for peripheral spondyloarthritis and for spondyloarthritis in general. Ann. Rheum. Dis..

[16] Probst C.M., Bompeixe E.P., Pereira N.F., de O. Dalalio M.M., Visentainer J.E., Tsuneto L.T., Petzl-Erler M.L. (2000). HLA polymorphism and evaluation of European, African, and Amerindian contribution to the white and mulatto populations from Paraná, Brazil. Hum. Biol..

[17] Reis P.G., Ambrosio-Albuquerque E.P., Fabreti-Oliveira R.A., Moliterno R.A., de Souza V.H., Sell A.M., Visentainer J.E.L. (2018). HLA-A, -B, -DRB1, -DQA1, and -DQB1 profile in a population from southern Brazil. HLA.

[18] John S.W.M., Weitzner G., Rozen R., Scriver C.R. (1991). A rapid procedure for extracting genomic DNA from leukocytes. Nucleic Acids Res..

[19] Lara-Armi F., Visentainer J., Alves H., Rocha-Lourdes M., Neves J., Colli C., Sell A. (2020). Optimization of HLA-B*27 ALLELE Genotyping by PCR-SSP. Clinics.

[20] Zacarias J.M.V., Sippert E.Â., Tsuneto P.Y., Visentainer J.E.L., e Silva C.d.O., Sell A.M. (2015). The Influence of Interleukin *17A* and *IL17F* Polymorphisms on Chronic Periodontitis Disease in Brazilian Patients. Mediat. Inflamm..

[21] Nemenqani D.M., Karam R.A., Amer M.G., Abd El Rahman T.M. (2015). Vitamin D receptor gene polymorphisms and steroid receptor status among Saudi women with breast cancer. Gene.

[22] Papadopoulou A., Kouis P., Middleton N., Kolokotroni O., Karpathios T., Nicolaidou P., Yiallouros P.K. (2015). Association of vitamin D receptor gene polymorphisms and vitamin D levels with asthma and atopy in Cypriot adolescents: A case-control study. Multidiscip. Respir. Med..

[23] Pepineli A.C., Alves H.V., Tiyo B.T., Macedo L.C., Visentainer L., de Lima Neto Q.A., Zacarias J.M.V., Sell A.M., Visentainer J.E.L. (2019). Vitamin D Receptor Gene Polymorphisms Are Associated With Leprosy in Southern Brazil. Front. Immunol..

[24] Torres T.M., Ciconelli R.M. (2006). Instrumentos de avaliação em espondilite anquilosante. Rev. Bras. Reumatol..

[25] Solé X., Guinó E., Valls J., Iniesta R., Moreno V. (2006). SNPStats: A web tool for the analysis of association studies. Bioinformatics.

[26] Barrett J.C., Fry B., Maller J., Daly M.J. (2005). Haploview: Analysis and visualization of LD and haplotype maps. Bioinformatics.

[27] Reis P.G., de Alencar J.B., Macedo L.C., Ambrosio-Albuquerque E.P., de Aquino J.S., Zacarias J.M.V., Tsuneto P.Y., Moliterno R.A., Sell A.M., Visentainer J.E.L. (2017). Cytokine gene polymorphisms in populations from Parana, Southern Brazil. Hum. Immunol..

[28] De Carvalho H.M.S., Bortoluzzo A.B., Gonçalves C.R., Da Silva J.A.B., Ximenes A.C., Bértolo M.B., Ribeiro S.L.E., Keiserman M., Menin R., Skare T.L. (2012). Gender characterization in a large series of Brazilian patients with spondyloarthritis. Clin. Rheumatol..

[29] Lin H., Gong Y.Z. (2017). Association of HLA-B27 with ankylosing spondylitis and clinical features of the HLA-B27-associated ankylosing spondylitis: A meta-analysis. Rheumatol. Int..

[30] Khan M.A. (2002). Update on spondyloarthropathies. Ann. Intern. Med..

[31] Syrbe U., Baraliakos X. (2018). Spondyloarthritis. Z. Rheumatol..

[32] Marwa O.S., Kalthoum T., Wajih K., Kamel H. (2017). Association of IL17A and IL17F genes with rheumatoid arthritis disease and the impact of genetic polymorphisms on response to treatment. Immunol. Lett..

[33] Shao M., Xu S., Yang H., Xu W., Deng J., Chen Y., Gao X., Guan S., Xu S., Shuai Z. (2020). Association between IL-17A and IL-17F gene polymorphism and susceptibility in inflammatory arthritis: A meta-analysis. Clin. Immunol..

[34] Zhao T., Ma C., Wang W., Zhao B., Xie B., Liu J. (2020). Common variants in IL17F gene contributed to the risk of hip osteoarthritis susceptibility in Han Chinese population. Int. J. Rheum. Dis..

[35] Bafrani H.H., Ahmadi M., Jahantigh D., Karimian M. (2019). Association analysis of the common varieties of IL17A and IL17F genes with the risk of knee osteoarthritis. J. Cell. Biochem..

[36] Choi B.G., Hong J.Y., Hong J.R., Hur M.S., Kim S.M., Lee Y.W., Choe Y.B., Ahn K.J. (2019). The IL17F His161Arg polymorphism, a potential risk locus for psoriasis, increases serum levels of interleukin-17F in an Asian population. Sci. Rep..

[37] Erkol Inal E., Görükmez O., Dündar Ü., Görükmez Ö., Yener M., Sağ Ş.Ö., Yakut T. (2015). The Influence of Polymorphisms of Interleukin-17A and -17F Genes on Susceptibility and Activity of Rheumatoid Arthritis. Genet. Test. Mol. Biomarkers.

[38] Weaver C.T., Harrington L.E., Mangan P.R., Gavrieli M., Murphy K.M. (2006). Th17: An Effector CD4 T Cell Lineage with Regulatory T Cell Ties. Immunity.

[39] Amatya N., Garg A.V., Gaffen S.L. (2017). IL-17 Signaling: The Yin and the Yang. Trends Immunol..

[40] Rossini M., Viapiana O., Adami S., Idolazzi L., Fracassi E., Gatti D. (2016). Focal bone involvement in inflammatory arthritis: The role of IL17. Rheumatol. Int..

[41] Ehrenfeld M. (2012). Spondyloarthropathies. Best Pract. Res. Clin. Rdheumatol..

[42] De Toledo R.A., de Toledo R.A., Camargo U., da Silveira Camargo A.V., Xavier D.H., Batista M.F., Carneiro O.A., Robles J.A., de Mattos C.C., Júnior O.R. (2015). HLA-B*27-Frequency of clinical signs in Brazilian patients with spondyloarthritis. Biomarkers Genomic Med..

[43] Gallinaro A.L., Ventura C., Barros P.D.S., Gonçalves C.R. (2010). Espondiloartrites: Análise de uma série Brasileira comparada a uma grande casuística Ibero-Americana (estudo RESPONDIA). Rev. Bras. Reumatol..

[44] Ribeiro S.L.E., de Campos A.P.B., Palominos P.E., Bortoluzzo A.B., da Costa M.A.C., de Oliveira Ribeiro T., Sampaio-Barros P.D. (2019). Different ethnic background is associated with distinct clinical profiles in the spondyloarthritides in the North and South of Brazil. Clin. Rheumatol..

[45] Deng S., He Y., Nian X., Sun E., Li L. (2020). Relationship between Vitamin D levels and pain and disease activity in patients with newly diagnosed axial spondyloarthritis. Int. J. Nurs. Sci..

[46] Kolahi S., Khabbazi A., Kazemi N., Malek Mahdavi A. (2019). Does vitamin D deficiency contribute to higher disease activity in patients with spondyloarthritis?. Immunol. Lett..

[47] Zhao S., Thong D., Duffield S., Goodson N. (2017). Vitamin D deficiency in axial spondyloarthritis is associated with higher disease activity. Arch. Rheumatol..

[48] Pokhai G.G., Bandagi S., Abrudescu A. (2014). Vitamin D levels in ankylosing spondylitis: Does deficiency correspond to disease activity?. Rev. Bras. Reumatol..

[49] Kocyigit B.F., Akyol A. (2018). Vitamin D levels in patients with ankylosing spondylitis: Is it related to disease activity?. Pakistan, J. Med. Sci..

[50] Blauvelt A., Chiricozzi A. (2018). The Immunologic Role of IL-17 in Psoriasis and Psoriatic Arthritis Pathogenesis. Clin. Rev. Allergy Immunol..

[51] Barnas J.L., Ritchlin C.T. (2015). Etiology and Pathogenesis of Psoriatic Arthritis. Rheum. Dis. Clin. N. Am..

[52] Jansen D.T.S.L., Hameetman M., Van Bergen J., Huizinga T.W.J., Van Der Heijde D., Toes R.E.M., Van Gaalen F.A. (2014). IL-17-producing CD4+ T cells are increased in early, active axial spondyloarthritis including patients without imaging abnormalities. Rheumatology.

[53] Gaston J.S.H., Jadon D.R. (2017). Th17 cell responses in spondyloarthritis. Best Pract. Res. Clin. Rheumatol..

